# Community providers’ intentions to use a parent-mediated intervention for children with ASD following training: an application of the theory of planned behavior

**DOI:** 10.1186/s13104-018-3879-3

**Published:** 2018-10-30

**Authors:** Brooke Ingersoll, Diondra Straiton, Karís Casagrande, Katherine Pickard

**Affiliations:** 1 Michigan State University, 316 Physics Rd., East Lansing, MI 48824 USA; 2 Department of Pediatrics, JFK Partners, University of Colorado, Anschutz Medical Campus, 13121 E. 17th Avenue, Aurora, CO 80045 USA

**Keywords:** Autism, Parent-mediated intervention, Provider training, Theory of planned behavior

## Abstract

**Objectives:**

The theory of planned behavior (TPB) suggests that attitudes, subjective norms, and perceived behavioral control influence intentions to perform a behavior, and that intentions predict behavior. The present studies examined whether the TPB is applicable to community providers’ use of a parent-mediated intervention for children with autism spectrum disorder (ASD) following introductory training and whether TPB constructs can be modified with training.

**Results:**

Study 1 demonstrated that community providers’ intentions to use the intervention post-training predicted their use of the intervention 6 months later [X^2^(1) = 8.03, p = .005]. Study 2 found that provider education (β = .23, t = 2.27, p = .025), attitudes (β = .21, t = 2.09, p = .039), and perceived behavioral control (β = .21, t = 2.15, p = .035) were all unique predictors of intentions. There was a significant increase in providers’ ratings of subjective norms (Z = − 2.46, p = .014) and perceived behavioral control (Z = − 7.36, p < .001) from pre- to post-training. Attitudes towards parent-mediated interventions were highly favorable pre-training and did not significantly increase. Results expand on previous findings and demonstrate the applicability of attitudes and perceived behavioral control in understanding community providers’ use of evidence-based practices for children with ASD.

## Introduction

Children with autism spectrum disorder (ASD) experience significant deficits in social interaction and communication, and the presence of restricted and repetitive behaviors [1]. They often require specialized intervention to learn new skills [2]; yet there is limited use of many evidence-based practices (EBPs) for these children in community settings [3, 4]. A better understanding of factors that influence community providers’ use of EBPs for children with ASD is important for the development of successful community training models.

Research in other fields suggests multiple factors that influence providers’ implementation of EBPs in community settings [5]. One such model is the theory of planned behavior (TPB) [6]. The TPB posits that an individual’s attitudes, subjective norms, and perceived behavioral control (PBC; degree of self-efficacy of performing a given behavior) predict intention to perform a behavior [6]. Intention, in turn, predicts actual behavior [7, 8]. This model has been used to understand and predict many health-related behaviors [9], and interventions aimed at improvements in health-related intentions have been associated with behavior change [10]. However, the TPB has only recently been examined in relation to the implementation of EBPs for children with ASD in community settings [11].

Fishman and colleagues demonstrated public school teachers who reported strong intentions to use visual schedules, an EBP for children with ASD, were significantly more likely to be observed using them with their students than teachers who reported weaker intentions, providing support for the role of intentions in predicting behavior [11]. Further, teachers reported significant variability in their intentions to use four different EBPs following training. This suggests that measuring community providers’ intentions to use EBPs following training may indicate the likelihood that that they will use them in their practice setting. Although this study provides evidence that community providers’ intentions to use a specific EBP for children with ASD can predict their actual use, it did not examine factors that influence providers’ intentions. Given the considerable variability in providers’ intentions to use various intervention strategies [11], it is important to examine variables that influence providers’ intentions and whether they can be modified with training.

The following two studies examine whether the TPB is applicable to community providers’ use of a manualized EBP for children with ASD following training. Specifically, it examines whether providers’ attitudes, subjective norms, and PBC predict their intentions to use Project ImPACT, an evidence-based parent-mediated intervention for children with ASD, and whether these variables are malleable with training.

## Main text

### Methods

Study 1 examined providers’ use of Project ImPACT 6 months after training, and assessed whether the magnitude of their intention to use Project ImPACT immediately following training predicted their use of it 6 months later. This study was necessary to examine implementation rates and validate the association between intentions and implementation in our training context [11]. Study 2 assessed whether providers’ attitudes, subjective norms, and PBC predicted their strength of intention to use Project ImPACT post-training, and whether introductory training positively influenced these factors.

Data for Studies 1 and 2 were derived from 11 Project ImPACT introductory training workshops conducted between 2014 and 2018 for community providers who worked with young children with ASD. Providers represented the many disciplines that provide services to young children with ASD in the community, including early intervention, special education, speech language pathology, psychology (ABA), social work, and occupational therapy. Providers also ranged in education level from some college to doctoral degree. See Table 1 for demographic information.

**Table 1:** Demographic information for participants

Characteristic	Study 1		Study 2	
	Percent (n = 57)	Mean (SD)	Percent (n = 92)	Mean (SD)
Workshop type				
3-day in-person workshop	33.3		19.6	
2-day in-person workshop with online tutorial	66.7		80.4	
Gender (% female)	94.7		93.5	
Age in years		36.47 (11.28)		35.18 (10.03)
Race/ethnicity				
White	91.2		91.3	
Black	1.8		2.2	
Asian	0		1.1	
Hispanic/Latino	0		2.2	
Multiracial	5.3		3.3	
Education				
Some college/specialized training	1.8		4.3	
4-year college degree	22.8		29.3	
Master’s degree	68.4		57.6	
Doctoral degree	3.5		8.7	
Occupation				
Social worker	12.3		7.6	
Speech-language pathologist	28.1		20.7	
Special education teacher	5.3		6.5	
Early intervention provider	12.3		12.0	
Psychologist	7.0		3.3	
Occupational therapist	14.0		8.7	
Graduate student	0		15.2	
Other	19.3		26.1	
Years experience working with children with ASD (years)				
< 1	7.0		12.0	
1–3	19.3		17.4	
4–6	22.8		21.7	
7–10	17.5		14.1	
11+	29.8		34.8	

Project ImPACT is a manualized parent-mediated intervention for young children with ASD that is based on best practices in early intervention for children with ASD. The curriculum uses effective adult learning and coaching strategies to teach parents to use naturalistic developmental-behavioral intervention strategies (NDBI) to promote their child’s social communication development [12].

The training workshop was delivered in either a 3-day in person or a 2-day in person plus online tutorial format. Both formats covered the same intervention and coaching content. The 3-day workshop presented the intervention strategies in a live lecture format on day 1 while the 2-day format presented this same information in an online tutorial completed prior to workshop. The rest of the workshops was identical, and involved a combination of didactic instruction, video review, small group discussion, case studies, and role play.

Workshop participants were asked to provide demographic information and complete a survey before and after the introductory training workshop. Demographic information included age, gender, race and ethnicity, education level, occupation, and years of experience working with children with ASD. Participants then received a follow-up survey by email 6 months later that inquired about their experience using Project ImPACT in their practice setting. Participants who completed the follow-up survey received a $5 gift card by email. All surveys were anonymous; participants were asked to generate a code that could be used to match their responses across time points. Of the 306 providers who attended the workshops, 125 (41%) produced viable data and gave consent for their data to be used in research.

Providers in Study 1 attended one of nine Project ImPACT introductory training workshops conducted between 2014 and 2017 (n = 276) and were sent a follow-up survey 6 months post training. Fifty-seven responded, for a 21% response rate. Respondents did not differ significantly on demographics from non-respondents. Providers in Study 2 attended one of five Project ImPACT introductory training workshops conducted between 2016 and 2018 (n = 110) and were asked to complete a survey of TPB variables immediately before and after the workshop. Ninety-two provided viable data (85% of attendees). Twenty-five participants in Study 2 also appeared in Study 1. See Table 1.

Participants in Study 1 completed measures of their intentions to use Project ImPACT immediately after the introductory training and their reported use of Project ImPACT 6 months post-training. Intentions were measured by asking participants to indicate the extent to which they intended to provide parent training in Project ImPACT on a scale of 1–7, with higher numbers indicating stronger intentions. The item stem was derived from social psychology and has strong content, face, and predictive validity for behavior [11, 13]. Reported use of Project ImPACT was measured by the question: *Have you implemented Project ImPACT?* Response options included: (1) Yes, I am implementing Project ImPACT as a group or individual parent training program; (2) Yes, I am implementing Project ImPACT, but in some other way that is not parent training; or (3) No, I am not currently using Project ImPACT in any way. For this analysis, response option 1 was coded as *Use of Project ImPACT* and response options 2 and 3 were coded as *No Use of Project ImPACT.*

Participants in Study 2 completed item stems to measure their attitudes, perceived norms, and PBC in training parents in Project ImPACT immediately before and after the training. Item stems were derived from social psychology and were rated on a Likert scale ranging from 1 (No, Definitely Not), to 7 (Yes, Definitely). Participants also rated their intention to provide parent training in Project ImPACT immediately post training on the same 7-point Likert scale as Study 1, with higher numbers associated with stronger intentions. See Table 2.

**Table 2:** Theory of planned behavior items

Construct	Item
Attitude	Parent-mediated intervention programs, like Project ImPACT, are an important part of a comprehensive intervention program for children with ASD
Subjective norm	Other providers I know use parent-mediated intervention programs like Project ImPACT
Perceived behavioral control	Do you feel you currently have the skills to conduct parent coaching in Project ImPACT in your work with families of children with ASD?
Intention	Please indicate the degree to which you intend to train parents to use Project ImPACT with their child with ASD

We used Spearman’s rho or Chi square analyses where appropriate to examine whether any provider demographics were associated with the outcomes of interest; any demographic variable that was significantly associated with the outcome of interest was included as a control variable in subsequent analyses. In Study 1, we used logistic regression to determine the effect of intentions at post-training on the likelihood that the participant used Project ImPACT 6 months later. In Study 2, we ran a hierarchical multiple regression to predict intention, using attitudes, subjective norms, and PBC. Education level was entered in Step 1 and the TPB variables in Step 2. Finally, we examined the degree to which the introductory training influenced the participants’ attitudes, subjective norms, and PBC using Wilcoxon Signed Rank tests.

### Results

In Study 1, 26 participants (46%) reported using Project ImPACT as a parent training program at follow-up. None of the demographic variables was associated with reported use of the program. The logistic regression model examining the relationship between post-training intentions and reported use of Project ImPACT at follow-up was significant, *X*^*2*^(1) = 8.03, *p *= .005. Increasing strength of intention at post training was associated with an increased likelihood of having used Project ImPACT as a parent training program 6 months following training, with a 2.1 times greater likelihood of using Project ImPACT for every 1 unit increase in intention.

In Study 2, education level was the only demographic variable associated with intention, r_*s*_= .30, p = .004. In the hierarchical multiple regression, education level was a significant predictor of intention in Step 1 (*F *= 7.38, *p *= .008). Adding the TPB variables significantly increased the fit of the model (*R*^*2*^ change = .09, *F *= 3.12, *p *= .03). In the final model, provider education level (β = .23, t = 2.27, p = .025), attitudes (β = .21, t = 2.09, p = .039), and PBC (β = .21, t = 2.15, p = .035) were all found to be unique predictors of intention. At pre-treatment, participants held highly favorable attitudes towards parent training (M = 6.55, SD = .76); attitudes post-training were similarly high (M = 6.66, SD = .65), and not significantly different from pre-training, *Z *= − 1.32, *p *= .19. Subjective norms increased significantly from pre-training (M = 4.23, SD = 1.45) to post-training (M = 4.63, SD = 1.52), *Z *= − 2.46, *p *= .014. PBC also increased significantly from pre-training (M = 3.65, SD = 1.67) to post-training (M = 5.32, SD = 1.01), *Z *= − 7.36, *p *< .001.

### Discussion

Fewer than half of the providers who attended the introductory training reported using Project ImPACT after training. This finding of low uptake is consistent with many other studies in the field [14] and suggests that a single training workshop in an EBP, such as Project ImPACT, does not ensure its use. Consistent with findings in other fields, Study 1 found providers’ intentions predicted their reported use of Project ImPACT 6 months later. Clearly, other factors beyond intentions also affect the use of an EBP [5]. However, our findings along with Fishman et al.’s [11] suggest that intentions are an important training outcome when trying to understand and increase providers’ use of EBPs for children with ASD in community settings.

Study 2 found partial support for other aspects of the theory of planned behavior; providers who expressed more positive attitudes towards parent-mediated intervention and providers who had higher PBC expressed stronger intentions to use Project ImPACT post training. This finding is consistent with other studies of health-related behaviors (e.g., smoking habits, seeking medical care) [9]. Surprisingly, we did not find an association between providers’ perception of subjective norms and providers’ strength of intention. However, this is not uncommon in studies of intention to perform health-related behaviors. In one meta-analysis [13], subjective norms were significant predictors of intentions in less than half of the studies, with regression coefficients for subjective norms being lower than those of attitudes and PBC. Additionally, our measure of subjective norms focused on the perceived behavior of other providers rather than perceived social pressure to perform a behavior [15]. It may be that subjective norms related to social pressure are more applicable for this population. Future research should examine the role of normative beliefs of community providers regarding EBPs for children with ASD.

We also examined whether our training increased favorable attitudes, subjective norms, and PBC. Providers held very favorable attitudes towards parent-mediated intervention prior to training; thus, our lack of a significant increase in provider attitudes is likely due to a ceiling effect. Although not as relevant in this training context, increasing positive attitudes towards an EBP may be particularly important for training in practices that are not viewed as favorably by community providers.

We found a significant increase in providers’ PBC in response to training in Project ImPACT. Given the relationship between providers’ PBC post training and their reported intention to use Project ImPACT, it appears that PBC or self-efficacy is a particularly important training outcome. Thus, community trainings that utilize instructional methods that maximize provider self-efficacy may be particularly important for increasing the use of EBPs. We also found an increase in providers’ subjective norms regarding parent-mediated intervention from pre to post training. Although we did not find a relationship between subjective norms and intention to use Project ImPACT, the fact that subjective norms can be changed with a brief training is of interest and may suggest a potential target for increasing providers’ use of EBPs in other contexts.

### Limitations

These data were collected as part of our community training efforts, which greatly limited the amount and type of data that could be collected. While we believe research in this context increases its ecological validity, it also introduces several limitations, including a low rate of follow-up data, reliance on provider self-report of program use, and use of single item measures of the TPB variables. Thus, future research should confirm and expand these findings by collecting more comprehensive data on TPB constructs [16] and provider use of Project ImPACT at follow-up, as well as formally testing the TPB within a single statistical model. In sum, our data suggest that the theory of planned behavior is generally applicable to community providers’ use of EBPs for children with ASD and can be used to guide the development of successful community training models.

## Abbreviations

ASD: autism spectrum disorder
TPB: theory of planned behavior
EBP: evidence-based practice
PBC: perceived behavioral control
